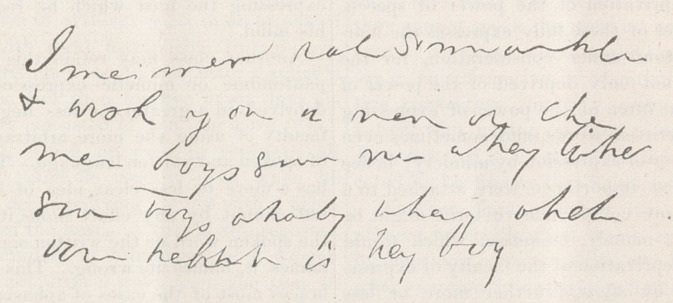# Aphasia

**Published:** 1872-01-15

**Authors:** J. B. Lyman

**Affiliations:** M. D. (Rockford)


### APHASIA.

BY J. B. LYMAN, M. D. (ROCKFORD.)

*Read before the Rockford Medical Association, and published at their request.*

The subject of this paper may be best introduced by a brief report of a case that occurred in my practice a few years since, and which has lost none of its interest by age. On the seventh of January, 1867, about six o'clock in the evening, I was called to see Mrs.——, then near the termination of her sixth pregnancy, and suffering at the time from a cold, with a very severe cough. I learned on my arrival that she was attacked while at the dinner table with a difficulty of speech and defect of vision. A note was also shown me which she wrote to send to her husband at his office to ask him to come home, as she was ill. This, together with the account given me of the symptoms during the afternoon, showed me at once that I had an exquisite case of aphasia, attended with agraphia and hemiopia; for she had been, and was still, unable at times to use the words which she intended. Wishing to say that her hand was numb, she said it was "nuff." Wishing to inquire, as nearly as I could judge, about the pain in the left side of her head, she asked why it "loved in her black." The agraphia was even more marked than the aphasia, as may be judged by the note which she attempted to write to her husband, of which a *fac simile* is here given. The note is plainly written, and in her usual handwriting; but it not only does not convey the meaning which she intended, but is destitute of any meaning whatever. The words "me," "men," and "boys," can be distinguished — the latter suggested perhaps by the circumstance that she wished to send her boys with the note. She made a second attempt, which consisted simply of the words "I do not know know know." She then abandoned the attempt, fully conscious that she had not written, and could not write, what she wished.

The hemiopia was distinct at the commencement of the attack, and continued until after my arrival. She could not see the left half (at her own right hand) of the face of a person sitting opposite to her at table. She complained also of severe pain in the left side of her head. The aphasia was not complete, as she used more than half the time the words that she wished. I decided upon venesection, and drew several ounces of blood, and all her symptoms were greatly relieved before the bleeding was discontinued. She miscalled some words after that, but would generally correct herself immediately. Sinapisms were ordered to be applied to the feet. I saw her again the same evening; there was still some aphasia and pain in the head, though they had much abated. A purgative of castor-oil was given, which operated in the night. At my visit on the eighth I found her quite relieved of all her symptoms except a slight pain in her head. On the 11th she was taken with labor pains, and was delivered on the morning of the 12th. The labor was regular and not severe, and she made a good recovery, without any return of the aphasiac symptoms, and there have been none since, though she has passed through another pregnancy. The discussion of the pathology of this case will be postponed to a subsequent part of this paper until after a brief consideration of that pathological condition, or rather symptom, which is now generally designated by the term *aphasia*.

Formerly the distinction between aphonia, or loss of voice, and aphasia, or loss of speech, was not sufficiently established. After this distinction was clearly seen and made, the loss of speech was at first designated by the word *alalia*. It was so called by Lordat in 1841. It was afterwards called *aphemia* by Broca in 1861. And finally it was denominated *aphasia* by Trousseau, the term which is now most generally in use. All of these terms have about the same meaning, and denote deprivation of the power of speech. But neither of these fully expresses the morbid symptom under consideration, for the patient is not only deprived of the power of speech, but often of the power of expressing ideas by written words, and sometimes even of the power of expression by mimicry; hence if any great importance were attached to a name, a more comprehensive term might be suggestive, namely, *asemasia*, which would denote a deprivation of the faculty of expressing ideas by *signs*, whether more or less arbitrary, as in speech and written language, or by imitation, as in mimitic acts. A patient suffering from this disordered condition has a more or less distinct idea of an object of thought, but is unable to express it — to embody it in any outward sign or symbol. The connection is cut off between the idea and its representative expression. A barrier is raised between the inner life of thought and the outer world. The expression of ideas by mimicry is a species of natural language, which is susceptible of cultivation, and may be made a source of entertainment, as in pantomimes or dumb shows. And we may assume this to be the last faculty of expression to be lost by disease. And yet in some cases of aphasia — or, if you please, of asemasia — the patient is deprived more or less completely of even this mode of expression. For example, Trousseau tried the following experiment with one of his patients named Paquet. He imitated before him the motions of a musician playing upon the clarionet, and asked the patient to do the same, which he did with perfect precision *by imitating him*. Trousseau waited a few moments and then asked him to make the motions of a man playing the clarionet, and although the patient knew well what instrument was intended, and probably had a clear conception of it in his mind, yet he found himself, generally, unable to embody that conception in so simple a mimetic act as that desired of him. Trousseau affirmed that this inability to express ideas by pantomime is found in many cases of aphasia. And this may be regarded as the highest degree of this morbid symptom, for the patient is deprived of all means of expressing the idea which he really has in his mind.

Another class may retain this faculty of pantomime or mimetic expression, and be deprived in a greater or less degree of the faculty of using the more arbitrary symbols of spoken and written language. The patient has a more or less clear idea of the object, but cannot by any effort *name* it, either by the spoken word or the written sign, or, if he names it, names it wrong. This class embraces most of the cases of aphasia, of which there are many interesting ones on record, exhibiting the symptom in a great variety of forms.

Approaching still nearer to the outer world, we find a class of patients who have clear conceptions of objects and of the words by which they are expressed, but may have lost the co-ordinating power by which the several nerves concerned in the complex act of speech are made to concur in the one result. The co-ordinating center or ganglion, if there be such, for the organs of speech, is impaired by disease. This class of patients may be able to write the word with ease, while they are utterly unable to speak it. Although this lesion is generally included under the term aphasia, still I think it would be better to exclude it, since it is manifestly a different faculty that is impaired, and dependent on a different portion of the nervous center. Still more must all cases of aphonia be excluded, where the patient has a clear conception of objects and of the words by which they are expressed, and the power of coordination complete, but one or more of the muscles concerned in phonation are paralyzed.

Limiting then the term aphasia to the deprivation, more or less complete, of the faculty of language, we find numerous cases reported manifesting this symptom in various degrees from absolute speechlessness to the simple misnaming of objects. There are cases in which no word is uttered, and yet the patient evidently makes a great effort to do so, and seems greatly annoyed at his want of success. A case is reported by Dr. Rinchenback in the hospital of Strasbourg, of an under-officer of artillery who, in an attack of aphasia with right hemiplegia, though evidently intelligent, could not utter a syllable for a week, and then for five or six days his vocabulary consisted of but one word ("*ja*," or "yes"), which he used in reply to all questions. And he used it even when he meant *no*, accompanying its use with a negative sign of the head. A case is also reported by Dr. Saunders, of Edinburgh, of a woman who did not utter a word from the time of her attack to the time of her death. Also a case by Dr. Hill, of Missouri, where the patient at times could not make an articulate sound, and at other times could utter only *yes* and *no*. And another case is reported by Dr. Addinell Hewson, of Philadelphia, of traumatic origin, where the patient was utterly unable to speak, except in a drawling attempt to say *yes sir*.

There is another large class of cases in which the patient can command but one or a very few syllables, which he uses on all occasions and in reply to all questions. Sometimes they are words not found in any language, and sometimes they are words in use, but to which the patient seems to attach no definite meaning. The vocabulary of one patient was limited for several hours to the one meaningless word *monomomontif*, and when he began to use other words he would give the first syllable correctly and then add the termination *tif*. For example, instead of *bon jour*, he would say *bon tif*. Another for eight hours could use no other words but *nasi bouse*, which he used alike in naming objects and in asking and replying to questions. And yet he seemed to recognize every object. The vocabulary of another for four months was limited to the word *cousise*, which he used on all occasions. Another had but one syllable at his command—*tan*—which he gave in answer to every question, usually repeating it, as "*tan, tan*." He was known in the hospital by the name of *Tan*. In another case the language of the patient was reduced to the one syllable *ta*, and yet the patient was quite intelligent. Others use words which are found in their language, as "oh fool," "my faith," etc. And, singular to say, some will use the word *yes* when they cannot speak the word *no*, and will indicate by a gesture whether they use the word *yes* in an affirmative or negative sense. In this connection we may mention another patient who, having occasion to use the word *four*, denoted it by the word *three*, and indicated by his fingers that he meant four.

Another class of cases will use longer phrases, especially those which are prompted by emotions, such as oaths, which are somewhat common in aphasia patients that have lost the power of speech more or less in other respects. Such phrases, indeed, are not so much language in its original office of naming our conceptions, as interjectional exclamations or explosions of feeling, and the aphasiac patient will use them when he is utterly incapable of using the same words separately as the substantive names of things. For example, one patient pronounced distinctly the phrase *it is no matter*, but could not possibly speak the word *matter* alone as a substantive. A very intelligent gentleman, a patient of Trousseau, affected with this disease, accidentally dropped his handkerchief, and a lady present picked it up and handed it to him. In his instinctive politeness he said "*merci*" (I thank you), but when requested immediately afterwards to repeat the word, he could not do it by any effort, although the word was pronounced before him.

There are many cases in which if the true word is suggested to the patient he will signify his assent by a gesture, although unable to speak the word himself. In other cases, if a word is suggested he will pronounce it like a parrot, and then repeat it in answer to every other question. We see this in the case of Trousseau's patient Marcou, a case interesting in many respects. When asked, "Are you not from Haute Soire?" he replied "Haute Soire." "What is your name?" "Haute Soire." "What is your business?" "Haute Soire." "But is not your name Marcou?" "Yes sir." "Is your name really Marcou?" "Yes." "What is your native place?" "Marcou."

There are, finally, many cases where the patient talks freely, and sometimes correctly, but is entirely unable at times to use the right word, substituting another word of an entirely different meaning in its place. We have an example of this in the case reported at the commencement of this article. We have, then this symptom exhibited in various degrees, and did time permit, many and curious examples could be given.

Aphasia is often, perhaps in most cases, attended with agraphia, or inability to express ideas by written as well as spoken words, both pointing to the same lesion, and, as I have said before, cases which do not exhibit both symptoms, should perhaps be excluded. An interesting case of agraphia is reported by Dr. Gairdner, of Edinburgh, where the patient was utterly unable to write his own name, but when the written name was placed before him, he could write it perfectly well, copying it apparently as he would copy a picture, the copy, however, partaking somewhat of the character of his own handwriting. In his unsuccessful attempt, knowing what the name is, you can perceive an illegible scrawl, an effort to write it. The specimen which I have shown you of our patient is perhaps as perfect an example of aphasiac writing as can be found; for though plainly and distinctly written, there is no meaning whatever conveyed by it, yet the patient was quite conscious of what she intended to write.

In this last respect cases of aphasia differ from each other, some being entirely conscious that they are not using, and cannot use, the words which they intend, and manifesting great annoyance at their failure, while others are utterly unconscious of having made any mistake. A lady suffering from this disease invited her friend who called upon her to a seat with this strange allocution: "*Pig, animal, deuce of a beast!*" Her son-in-law, who was present and who was a physician, found it necessary to interpret to the visitor that his mother-in-law invited her to be seated, she being entirely unconscious of having used any but the politest language. There is another case reported of a Dr. Spalding, of Berlin, who, while in this condition, had occasion to write a receipt, and supposed he had written it correctly, but when he recovered found to his astonishment that instead of writing *Received fifty dollars for one-half year's interest*, he had plainly written, *Received fifty dollars for the sanctification of the Bric.* A case is reported in the *American Journal of the Medical Sciences*, of a lady in Natchez who while in this state evinced great astonishment that she was not understood, and yet her conversation was quite unintelligible. Probably in most cases, however, the patient is conscious of his inability to express his ideas correctly.

In regard to accompanying symptoms, there have been cases in which the defect of speech was the only marked indication of disorder of the nervous centers. In other cases there has been numbness of one side. But most cases have been attended with hemiplegia, and what is very remarkable, with hemiplegia of the right side. This latter circumstance has attracted much attention, and given rise to much discussion in cerebral physiology. I have found no case reported that was accompanied with hemiopia except the one which I have reported at the commencement of this article.

The pathology of these cases has attracted much attention, and been the subject of various opinions. Here we cannot enter much into detail, in reporting particular cases which are found in our works and journals. It may suffice to state that in most of the cases attended with hemiplegia, and where autopsies have been made, there has been found more or less extended lesion of the left cerebral hemisphere, corresponding to the right hemiplegia. These lesions have been cerebral softening clots, from hemorrhage, tumors and mechanical injury. From the indications furnished by these lesions, attempts have been made to locate the faculty of language, and they have been carefully studied with this view. The three most important theories have been those of Bouillaud, who locates the faculty in the anterior lobes of both hemispheres, that of Dax, who locates it in the left hemisphere, and that of Broca, who limits it to the posterior part of the third frontal convolution of the left side. Strange as it may seem, this last view has much in its favor, and has been well received by some. But for one I could not adopt it, even if there were no decisive facts to be adduced against it. In a double organ so nearly symmetrical as the brain, whose median line corresponds with the median line of the body, it is so contrary to all the analogy of nature that corresponding parts on each side should have different functions, that I could not receive it except on the most convincing evidence. But while most of the facts are in favor of the localization in the left hemisphere, yet there are some cases which militate against it, and still more against its location exclusively in the third frontal convolution of that side. Two cases are reported by Abercrombie where there was loss of speech with *left* hemiplegia, in one of which there was cancer, and in the other tubercle in the *right* hemisphere. And many cases are reported by Andral of loss of speech without injury of the anterior lobes. But it must be remembered in reading these older writers, that loss of speech is not synonymous with aphasia, which is the loss of language or the idea of speech. Some of these cases might have arisen from paralysis or loss of co-ordinating power. The subject of aphasia had not then been studied as carefully as it has been more recently. There are, however, undoubted cases of aphasia which go to disprove the views of Broca. In the case of the patient Marcou, which we have before referred to, the aphasia was undoubted, and yet the hemiplegia was on the left side, and consequently the lesion was on the right side. But as this case did not terminate fatally, it has been argued that there may have been a lesion on the right side, producing the hemiplegia, and another on the left side, producing the aphasia. Trousseau, in reply, admits that multiple lesions are not very uncommon, but thinks they are usually found in severe apoplectic cases, and in cases of extensive traumatic injury. But this was a very mild case, and what is more to the point, both symptoms were developed simultaneously. There is another case of great weight on this question in the St. George's Hospital reports, as given in the *American Journal* for July, 1870, in which the hemiplegia was on the left side, and the lesion was the entire destruction of the island of Reil on the right side. And there have been many cases of aphasia where Broca's convolution, so called, was not injured; one in particular is reported, where Broca himself was present at the autopsy. There is a case reported by Echeverria in the *Medical Record* for March 1st, 1869, and in the *American Journal* for April of the same year, showing that there may be lesion of that convolution without aphasia. It has been thought there was no such case on record. On the other hand it may be said in favor, so far as it goes, of Broca's theory, that there have been undoubted cases of lesion of the corresponding convolution on the *right* side without loss of speech. In fact, it must be admitted that aphasia, as a rule, results from lesion of the left side of the brain, so that in a sense the faculty of language is located on the left side. But in what sense? An explanation of this has been given, which I think has much in its favor. In the case just related, of left hemiplegia with aphasia, from the St. George's Hospital reports, the patient was at the same time ambidextrous, and indeed, rather inclined to use his left hand. And so, as we use both hands, but one better than the other, so the faculty of language may be more completely educated on one side than on the other; and when, after an attack of aphasia, the patient again *learns* the use of language, as this patient did, we may assume that he educates himself to use the other side of the brain for language, as he does for the use of his remaining hand, after losing the one most commonly used. This aphasiac patient was exceptionably hemiplegic on the left side; but he was also, exceptionably, left-handed. This view is strengthened by the theory, if it be true, that the left side of the brain is developed earlier than the right. Further investigation and new cases may hrow more light upon this much disputed question.

A word in respect to the causes of this singular affection. Cases are reported of traumatic origin. Many are doubtless produced by Emboli in cerebral arteries, especially the left middle cerebral. Others are caused probably by an atheromatous condition of the arteries. Cases are reported produced by the bite of venomous serpents, the virus producing, it is thought by some, spasms of the middle cerebral. Other cases are referred to malaria, as in the case of a patient in the East Indies, who was attacked in the autumn three years in succession. Dr. Mack of East Portland, Oregon, formerly of Illinois, relates his own case in the *Medical and Surgical Reporter* for October 28th, of this year. He was surgeon in the army, but resigned on account of congestive chills, with paralysis of the *left* arm. One year afterward, he was attacked with aphasia while giving directions to a patient. The attack lasted about an hour. On the following day he had a similar attack at the same hour. Five years later he had two more attacks with an interval of six days. The first attack, as before, was while giving directions in regard to medicine, and it continued eleven hours. During the last attack, which continued four hours, he felt symptoms of paralysis in *both* hands. This case was probably of malarial origin, though possibly it was hereditary; for he states that his father has been paralyzed on one side for ten years. There are also cases referred to Bright's disease, to venereal excess and to syphilis.

In the treatment of these cases but little has been established. The milder cases are undoubtedly benefited by venesection. This was the case with a distinguished physician in Paris, who was affected with this disease. He made signs that he wished to be bled, and he was immediately benefited by the operation. And certainly, in the case that I have reported, the patient was speedily relieved by the same treatment. A case is reported by Trosseau that was much benefited by leeches to the arms. No special treatment can be indicated for the severe cases attended with hemiplegia. Many cases however have recovered; and many patients have, by laborious effort, been taught again the use of language.

It is a question of much interest pathologically, and in a medico-legal point of view, how far the intelligence is affected in this disease. While it is admitted that this is impaired, except in mild cases of brief duration, yet unquestionably it is to a great extent preserved. Persons in this condition have been able to attend to their business, and to play at games with their usual skill; and that too at games of cards that require very accurate memory. Distinguished physicians, while in this condition, have been able to study and analyze their own symptoms, and were possessed of their usual intelligence, certainly to a great extent; as was the case with Dr. Spalding of Berlin and a distinguished professor in Paris. This was eminently true in the case of Prof. Lordat of Montpelier. By his own account, though completely aphasiac, his mind was clear. "I experienced," he says, "no embarrassment in the exercise of thought. Accustomed as I had been for a long time, to the labor of a professorship, I was inwardly happy, to be still capable of arranging in my mind the principal propositions of a lecture, and to experience no difficulty in changing the order of ideas as I pleased." He could combine, he says, abstract ideas, and readily distinguish them, without any word to express them, and without thinking of that expression the least in the world. But even in his case, the mental faculties must have been more or less impaired; for previous to the attack he possessed, in a remarkable degree, the faculty of lecturing without notes, which he was not able to do afterwads, nor could he ever memorize a lecture, but was obliged to read it from the manuscript.

The question of the legal responsibility of these patients is one of some difficulty. And yet in the cases that I have related, and in many others, even in those of still greater severity, the patients generally exhibit sufficient intelligence, it seems to me, to render them legally responsible, provided means of communication can be contrived. They have not lost the faculty of memory, but the *memory of words*. In other respects they may be intelligent. Each case however, will probably have to be decided on its own merits; at least until the general question can be more fully settled.

The case of Prof. Lordat raises a metaphysical question of no small interest. He says that, when in this state, he reflected upon the Christian doxology, *Glory to the Father, Son and Holy Ghost*, but it was impossible to recall a single word composing the phrase. He could combine abstract ideas without a single word at his command to express them. The soul of thought he had, but the body was gone. The question is, can mental actions, processes of thought, be carried on without the use or the thought of language? If we accept the statements of the professor of Montpelier, they can. It has been said however, that he was an idealist, and therefore interested in that direction. That may be; and we all know how much preconceived opinions may influence us in the interpretation of facts. We are so habituated to the use of words in carrying on our mental processes, that it is impossible to decide this question from our own consciousness. It is possible however, that much light may be thrown upon it in future by a clear observation of cases of aphasia that may occur.

A word in closing, in regard to the pathology of the case reported at the commencement of this paper. In so mild a case and one which recovered so soon and so completely, we cannot assume any organic lesion of the brain. An Embolus could have scarcely caused the attack, unless it was speedily dissolved, or so small as to admit partially of the passage of the sanguineous current. As she was suffering from a severe cough at the time under the complication of advanced pregnancy, I think we may attribute the attack to congestion. That the trouble was in the left side of the brain, is evident from the pain on that side of the head, from the numbness of the right arm, and from the hemiopia; for it will be recollected that she could not see the left side of the face of a person sitting opposite to her at table. And if we follow the rays of light, proceeding from that side of the face, we shall find that they lead to the left side of the brain of the patient. Supposing the axes of her eyes to be directed to the middle line of the face opposite to her, the rays of light will pass from the left side of that face to the left side of the retina of each of her eyes. The impression will then pass from the outer half of the retina of her left eye along the outer side of the left optic nerve, to the chiasma, and thence, without crossing, it will pass along the left root of the chiasma to the left side of the optic tholamus and the corpora quadrigemina, and how much farther in the left hemisphere I believe has not yet been determined. The impression on the inner or left side of her right eye will pass along the inner side of the right optic nerve (leaving out of view the commissural fibers) to the chiasma, and then crossing to the left side, will pass with the impression from the left eye to the left side of the brain. But as the left side of the brain is suffering from congestion, the impression is not perceived, and therefore the left side of the face opposite to her was not seen. This case might be cited as supporting, in some degree, the opinion of Broca, or at least that of Dax, that the faculty of language is located in the left hemisphere.

---

Mortality of Small-Pox in Paris.—From July, 1869 to June, 1871. there were 13,614 deaths in Paris from small-pox, of which 1,800 were soldiers. In the civil hospitals there was one death to three cases, and in the military hospitals one to six.—*The Clinic.*

## Figures and Tables

**Figure f1:**